# Spittlebugs as vectors of *Xylella fastidiosa* in olive orchards in Italy

**DOI:** 10.1007/s10340-016-0793-0

**Published:** 2016-07-12

**Authors:** Daniele Cornara, Maria Saponari, Adam R. Zeilinger, Angelo de Stradis, Donato Boscia, Giuliana Loconsole, Domenico Bosco, Giovanni P. Martelli, Rodrigo P. P. Almeida, Francesco Porcelli

**Affiliations:** 1 Department of Soil, Plant and Food Sciences, University of Bari Aldo Moro, Bari, Italy; 2 Institute for Sustainable Plant Protection, National Research Council (CNR), Bari, Italy; 3 Department of Environmental Science, Policy and Management, University of California, Berkeley, CA USA; 4 Department of Agriculture, Forestry and Food Sciences, University of Turin, Grugliasco, Italy

**Keywords:** Emerging diseases, Plant pathogenic bacteria, Auchenorryncha, Aphrophoridae

## Abstract

The recent introduction of *Xylella fastidiosa* in Europe and its involvement in the Olive Quick Decline Syndrome (OQDS) in Apulia (Salento, Lecce district, South Italy) led us to investigate the biology and transmission ability of the meadow spittlebug, *Philaenus spumarius*, which was recently demonstrated to transmit *X. fastidiosa* to periwinkle plants. Four xylem-sap-feeding insect species were found within and bordering olive orchards across Salento during a survey carried out from October 2013 to December 2014: *P. spumarius* was the most abundant species on non-olive vegetation in olive orchards as well as on olive foliage and was the only species that consistently tested positive for the presence of *X. fastidiosa* using real-time PCR. *P. spumarius*, whose nymphs develop within spittle on weeds during the spring, are likely to move from weeds beneath olive trees to olive canopy during the dry period (May to October 2014). The first *X. fastidiosa*-infective *P. spumarius* were collected in May from olive canopy: all the individuals previously collected on weeds tested negative for the bacterium. Experiments demonstrated that *P. spumarius* transmitted *X. fastidiosa* from infected to uninfected olive plants. Moreover, *P. spumarius* acquired *X. fastidiosa* from several host plant species in the field, with the highest acquisition rate from olive, polygala and acacia. Scanning electron microscopy (SEM) revealed bacterial cells resembling *X. fastidiosa* in the foreguts of adult *P. spumarius*. The data presented here are essential to plan an effective IPM strategy and limit further spread of the fastidious bacterium.

## Key message

- *Philaenus spumarius* is a vector of *Xylella fastidiosa*, although transmission of the bacterium to olive with naturally infected spittlebugs has not been demonstrated.
- The main goal of this work was to test the vector transmission of *Xylella fastidiosa* to olive.
- We found that *X*. *fastidiosa* is transmitted by *P.* *spumarius* between olive plants. Three other xylem-sap feeders occurring in olive orchards tested negative for *X*. *fastidiosa*.
- In 2014, the first infective *P. spumarius* were collected on olive canopies, with infectivity increasing gradually from May throughout the end of August.
- Our findings are essential for effective management of *X. fastidiosa*.

## Introduction

Human activities, especially the speed and volume of transportation, have accelerated the global expansion of invasive species because of a breakdown of natural barriers to dispersal, so much so that the distribution of invasive species appears to be restricted primarily by climatic factors (Capinha et al. 2013). One activity highly impacted by invasive species is agriculture, where crop diversity has become gradually more homogeneous at the global scale (Khoury et al. 2014), leading to a suite of shared pests and diseases. Therefore, it is not surprising that some of the major current and future challenges to agriculture gravitate around the potential risks associated with the introduction of invasive species into new regions where they are absent.

The recent establishment of the vector-borne bacterium *Xylella fastidiosa* in the Salento peninsula (southern Italy) (Saponari et al. 2013) highlights the risks associated with the unintended introduction of organisms into new regions. The currently available phylogenetic data indicate that the invasive strain (named CoDiRO) belongs to the *X. fastidiosa* subspecies *pauca* and was possibly introduced from Costa Rica (Giampetruzzi et al. 2015) via infected ornamental plant material, which has recently been shown to be an important pathway for the long-distance dispersal of *X. fastidiosa* (EFSA 2015). Because *X. fastidiosa* vectors are present throughout the Mediterranean basin (EFSA 2015), and this bacterium colonizes several crop species of economic and cultural importance (e.g., grapevine, citrus, almond) (Hill and Purcell 1997), the threat due to its introduction to Europe is significant.

Despite the risks represented by the introduction of *X. fastidiosa* into Italy, it is difficult to infer how fast or widely the bacterium will spread in the region or how to best manage infected areas and limit bacterial dispersal. For the best-studied disease systems caused by *X. fastidiosa*, Pierce’s disease of grapevines in the USA and citrus variegated chlorosis in Brazil, nearctic and neotropic sharpshooter leafhoppers (Hemiptera, Cicadellidae) are the epidemiologically relevant vectors (Redak et al. 2004). However, in Europe this group is poorly represented, whereas spittlebugs (Hemiptera, Cercopoidea) are the dominant group of potential *X. fastidiosa* vectors (EFSA 2015). Spittlebugs have been known to transmit *X. fastidiosa* since the 1940s (Severin 1950), but only a limited number of studies have addressed the role of these insects on pathogen spread (Severin 1950; Purcell 1980; Sanderlin and Melanson 2010). Although there is no documented vector species-*X. fastidiosa* specificity for transmission, and all spittlebugs should be considered potential vectors until proven otherwise (Frazier 1965; Almeida et al. 2005), there are significant knowledge gaps on the biology of *X. fastidiosa* transmission by this group of insects.

*Xylella fastidiosa* is associated with disease symptoms in olive elsewhere. Krugner et al. (2014) reported that *X. fastidiosa* isolates belonging to the subspecies *multiplex* were inconsistently associated with olive leaf scorch symptoms in California, but failed to fulfill Koch’s postulates under greenhouse conditions with isolates from either ssp. *multiplex* or ssp. *fastidiosa*. An association of *X. fastidiosa* ssp. *pauca* with olive scorch in Argentina and Brazil has also been reported (Haelterman et al. 2015; Della Coletta-Filho et al. 2016). In addition, Krugner et al. (2014) demonstrated that a sharpshooter vector species could transmit isolates of the ssp. *multiplex* and *fastidiosa* from almond to olive trees. Saponari et al. (2014a) reported the first finding of field-collected *Philaenus spumarius* L. (Hemiptera: Aphrophoridae) positive by PCR for *X. fastidiosa*; the authors carried out a first transmission experiment with field-collected meadow spittlebugs from Gallipoli (Salento region, Apulia) caged in groups of 8–10 per plant on five periwinkle (*Catharanthus roseus*) plants and seven olive (*Olea europea*) plants for an Inoculation Access Period (IAP) of 96 h. Eventually, two out of five periwinkles tested positive for *X. fastidiosa*, whereas transmission to olive was not achieved. Moreover, the authors tested two Auchenorryncha species collected from November 2013 to January 2014, *Euscelis lineolatus* Brullè (Hemiptera: Cicadellidae) and *P.spumarius*, for *X. fastidiosa* by real-time PCR; only the latter tested positive for the bacterium. Although these preliminary data should be considered very important, the authors themselves suggested that further investigation is required, especially given i) the lack of *P. spumarius* transmission of *X. fastidiosa* to olive and (ii) that a survey of the candidate vector was limited to 3 cold months when most of the biological cycle of the species has to be considered concluded. Therefore, we performed a series of experiments and field sampling to gain insights into the candidate vectors' possible role in the *X. fastidiosa* epidemiology in the region. The overall goal of this work was to generate initial information on the role of candidate vectors, especially *P. spumarius,* in *X. fastidiosa* spread in the Apulia epidemic.

## Materials and methods

### Survey of xylem-sap feeding species in and adjacent to olive orchards

**Fig. 1:**
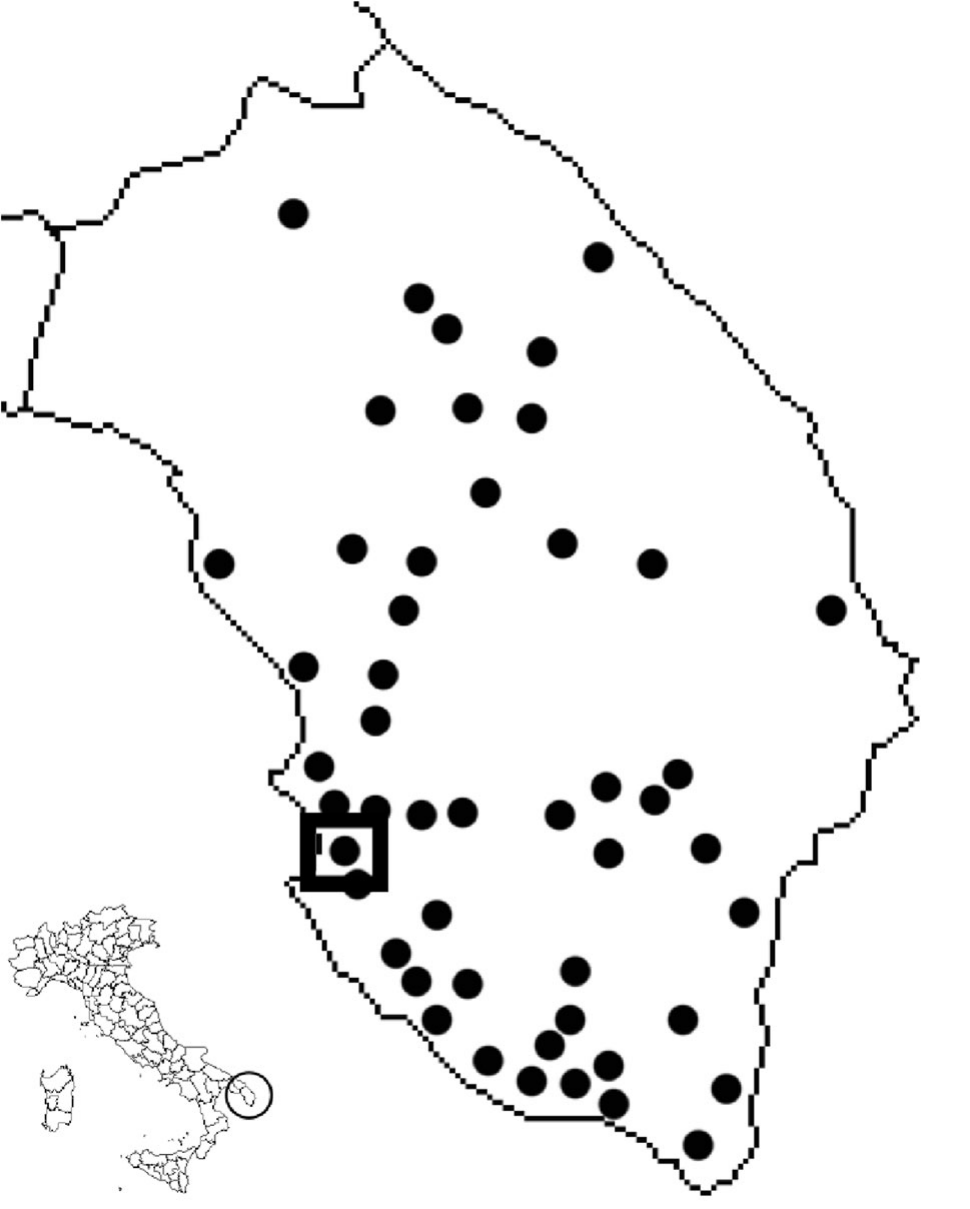
Field sites surveyed to determine the composition of a potential *Xylella fastidiosa* vector community. Site with square indicates the location where vector abundance and infectivity were determined. Inset: map of Italy, with the studied region circled. The maps were generated by authors using R version 3.2.0

A survey of xylem-sap feeders in olive orchards was carried out from October 2013 to December 2014 on 48 sites in Lecce district, mainly around the *X. fastidiosa* hotspot in Gallipoli (Fig. 1). While the six sites used in the study of Saponari et al. (2014a) were also included in our study, no data from the cited paper are included in our analysis. We visited every site at least twice, once in spring-summer and once in the fall. Four sites in the municipalities of Gallipoli and Alezio (Fig. 1) were visited every other week. In order to protect the anonymity of participating farmers, a detailed geographic location of each site cannot be made publically available. The study was carried out on private land with owner permission (no specific permits were required); the field survey did not involve endangered or protected species. The insects were collected with a sweep net continuously for 1 h per field per each collection date on olive trees, weeds and plants other than olive within and surrounding the orchards. Samples were stored in 75 % ethanol and brought to the laboratory for identification following Ossiannilsson (1981), Holzinger (2003), Ribaut (1952) and Della Giustina (1989). Furthermore, a collection of Auchenorryncha (insects and slide-mounted genitalia) used for this research has been taxonomically identified according to the reported references and is available at the University of Bari (DiSSPA, sezione Entomologia e Zoologia, via Amendola 165/A, 70125 Bari, Italy).

We used real-time PCR to detect the presence of *X. fastidiosa* in all the xylem sap feeders collected during each visit. At least 50 % of the insects were tested individually; the remaining ones were pooled in groups of four to five individuals of the same species. Moreover, at least 50 % of the Auchenorryncha other than xylem-sap feeders (mainly Cicadellidae as well as Fulgoromorpha Issidae and Cixiidae) collected in each site was screened by real-time PCR using individual specimens. DNA was recovered following Marzachì et al. (1998) and amplification of *X. fastidiosa* carried out using the primer set described by Harper et al. (2010). Samples yielding positive reactions were randomly selected and the purified PCR amplicons subjected to sequence analysis. All the samples confirmed the identity of the amplified DNA as *X. fastidiosa* (data not shown).

### Assessment of *Philaenus spumarius* abundance, host plant shifting and infectivity

From October 2013 to December 2014, we surveyed populations of *P. spumarius* (Hemiptera, Aphrophoridae) in a 1 ha. *X. fastidiosa*-infected olive orchard in the Gallipoli area (N 40 012704; E 018 05341). From October 2013 to May 2014, once every 15 days, weeds were swept continuously for 1 h during each visit. From May to the end of October 2014, weekly, we began to sweep olive canopies and suckers directly, in addition to weeds and plants other than olives present in the field: the main goal was to monitor changes in the relative abundance of adult *P. spumarius* on weeds and olive trees. Each sample consisted of ten sweeps performed on single olive plants or on wild plants, including weeds, within and at the boundaries of the orchard. The overall content of the sweeping net after the ten sweeps was emptied in a plastic bag properly labeled with the sweeping date and the swept plant, sealed, stored in a cool-box and brought to the laboratory (located into the infected area). Once in the laboratory, the *P. spumarius* were identified and counted, and the relative abundance on olive trees and hosts other than olive was calculated by dividing the number of spittlebugs collected from the total number of samples swept on the host during the 1 h sampling for the total number of samples (bags) collected on that host. *P. spumarius* collected in the middle of each month were tested individually by real-time PCR as reported above in order to assess infection prevalence in individuals collected from olive plants and weeds, i.e., the percentage of insects harboring the bacterium versus the total number of collected insects on different hosts during the season. During the November–December 2014 period, monitoring was performed once every 2 weeks.

### Olive-to-olive vector transmission of *X. fastidiosa*

We sought to test whether the two dominant xylem sap-feeding species, *P. spumarius* and *Neophilaenus campestris*, could successfully transmit *X. fastidiosa* between olive trees. From May to July 2014, we collected 20 *Neophilaenus campestris* and 20 *P. spumarius* individuals weekly by sweeping olive orchards in an *X. fastidiosa*-free area in Brindisi district for a total of about 400 insects; all these insects tested negative for *X. fastidiosa* by real-time PCR. This assessment verified that our subsequent collections from these sites would likely be free of *X. fastidiosa.* In July 2014, *N. campestris* and *P. spumarius* adults were collected from the same fields and brought to an infected olive orchard in the Gallipoli area to be caged on OQDS-symptomatic olive branches (testing positive for *X. fastidiosa*) for a 4-day acquisition access period (AAP). All *N. campestris* were caged together on one symptomatic branch, while *P. spumarius* individuals were divided into three groups, each group caged on a different plant previously testing positive for *X. fastidiosa* by real-time PCR. After the AAP, insects were taken back to the laboratory, separated in groups of individuals and allowed a 4-day IAP on self-rooted olive plants (*Olea europaea*), cultivar Coratina or periwinkle plants (*Catharanthus roseus*), which were maintained in an insect-proof greenhouse (Table 3). One olive plant and one periwinkle caged devoid of insects served as control. Insects that died during the AAP or IAP were stored in 75 % EtOH and PCR-tested for the presence of *X. fastidiosa* as described above. Three months after insect inoculation, inoculated and uninfected control test plants were tested for the presence of *X. fastidiosa* according to Loconsole et al. (2014).

### Role of host plant species on *X. fastidiosa* vector transmission

To better understand the role of the host plant on the ecology of the infections, *X. fastidiosa*-positive plant species from the Gallipoli and Alezio municipalities were used as pathogen sources for vector acquisition. In addition to olive, numerous plant species have been shown to be colonized by *X. fastidiosa* in southern Apulia, including almond (*Amygdalus communis*), oleander (*Nerium oleander*), cherry (*Prunus avium*), myrtle-leaf milkwort (*Polygala myrtifoli*a), coastal rosemary (*Westringia fruticosa*), acacia (*Acacia saligna*) and broom (*Spartium junceum*) (Saponari et al. 2013, 2014b). We investigated acquisition from acacia, broom, olive, almond, cherry, oleander, periwinkle and polygala. We identified suitable plants of each species that were confirmed by real-time PCR to be infected with *X. fastidiosa* for these experiments. No *Westringia* spp. plants were available for the experiment. One periwinkle infected by *P. spumarius* collected in Gallipoli at the beginning of June and maintained in a growth chamber was used as a positive control. In September 2014, *P. spumarius* presumably free from the bacterium were collected from the same *X. fastidiosa*-free area in Brindisi as described above. Twenty *P. spumarius* adults were caged as one group for a 96-h AAP on each infected host plant species under field conditions. In addition, five *P. spumarius* were caged on an uninfected acacia plant used as a negative control. At the end of the AAP, five or six *P. spumarius* were randomly selected from each host and transferred singly to periwinkle plants for a 96-h IAP in a greenhouse. Then, the IAP spittlebugs were stored in 75 % EtOH for real-time PCR assays. One month after the IAP, recipient periwinkle plants were tested for the presence of *X. fastidiosa* by real-time PCR (Loconsole et al. 2014).

### Scanning electron microscopy of the foregut of spittlebugs

To search for *X. fastidiosa* in *P. spumarius* foregut lumen, ten *P. spumarius* collected from diseased olive trees in Gallipoli in September 2014 were prepared according to Almeida and Purcell (2006), except that samples were fixed overnight in 4 % (vol/vol) glutaraldehyde in 0.05 M cold (4 °C) phosphate buffer (Pb) at pH 7, instead of cacodylate buffer, and observed by a Hitachi TM3000 low-pressure scanning electron microscope.

### Statistical analysis

We used repeated measures analysis of variance (ANOVA) to test for a difference between olives and weeds in (1) *P. spumarius* densities and (2) the proportion of collected *P. spumarius* infected with *X. fastidiosa*. In both tests, the source plant (olive or weeds; *n* = 8 for each level) was included as a fixed effect, and the date of collection was included as a repeated-measures random effect (Pinheiro and Bates 2000). In the first test, densities per sweep were used as the response to control for differences in the sampling effort between olives and weeds; standardized densities were then square-root transformed to meet assumptions of ANOVA—namely, that the error variance is constant and the response variable is normally distributed (Oehlert 2000). For the second test, the proportion of insects infected was arc-sine square-root transformed to meet assumptions of ANOVA.

We also tested for differences between olive and other host plants in *X. fastidiosa* acquisition and inoculation rates by *P. spumarius* on periwinkle. Here logistic regression was used with infection status as the binary response variable and plant species—the source plant for the acquisition experiment and test plant for the inoculation experiment—as the sole fixed-effect explanatory variable. No acquisition or inoculation occurred for some plant species; this resulted in all zeros for some plant species—called quasi-complete separation of factor levels in the statistics literature. Standard methods of logistic regression cannot estimate coefficients of factor levels in cases of complete or quasi-complete separation. As a remedy, we used logistic regression with Firth’s bias correction (Heinze & Schemper 2002). All analyses were conducted in R 3.2.0 (R Core Team 2015); the nlme package was used for the repeated-measures ANOVAs (Pinheiro et al. 2014) and the logistf package for Firth’s logistic regression (Heinze 2013).

## Results

### Xylem-sap feeding species found in or adjacent to olive orchards

In all the surveyed olive fields (Fig. 1), only two spittlebug species were collected on olive, *P. spumarius* and *N. campestris*. One cicada species (*Cicada orni*) was observed in olive orchards from June to August. We did not quantify cicada abundance on olive, since such data on cicadas require a dedicated study. From October 2013 to December 2014, the spittlebug population on olive plants was almost exclusively composed of *P. spumarius* (98.56 % of the overall spittlebug population collected from olive plants); *N. campestris* (1.44 % of the overall spittlebug population) was rare. Only 1 out of about 200 *N. campestris* that had been collected in July 2014 tested positive for *X. fastidiosa*; the single individual positive for the bacterium was collected on an oleander plant. A third spittlebug, *Cercopis sanguinolenta*, was collected on weeds bordering olive orchards from March to early May; none of the 31 *C. sanguinolenta* collected tested positive for *X. fastidiosa*. *C. orni* were collected mainly on olive trees; of the 54 adults and 18 *exuviae* tested, none was *X. fastidiosa* positive. Twenty-four *E. lineolatus* found on weeds, mainly *Mercurialis annua* at the border of the fields, collected from October to December 2014, tested negative for *X. fastidiosa*.

### Preliminary assessment of *Philaenus spumarius* abundance, host plant shifting and infectivity

**Fig. 2:**
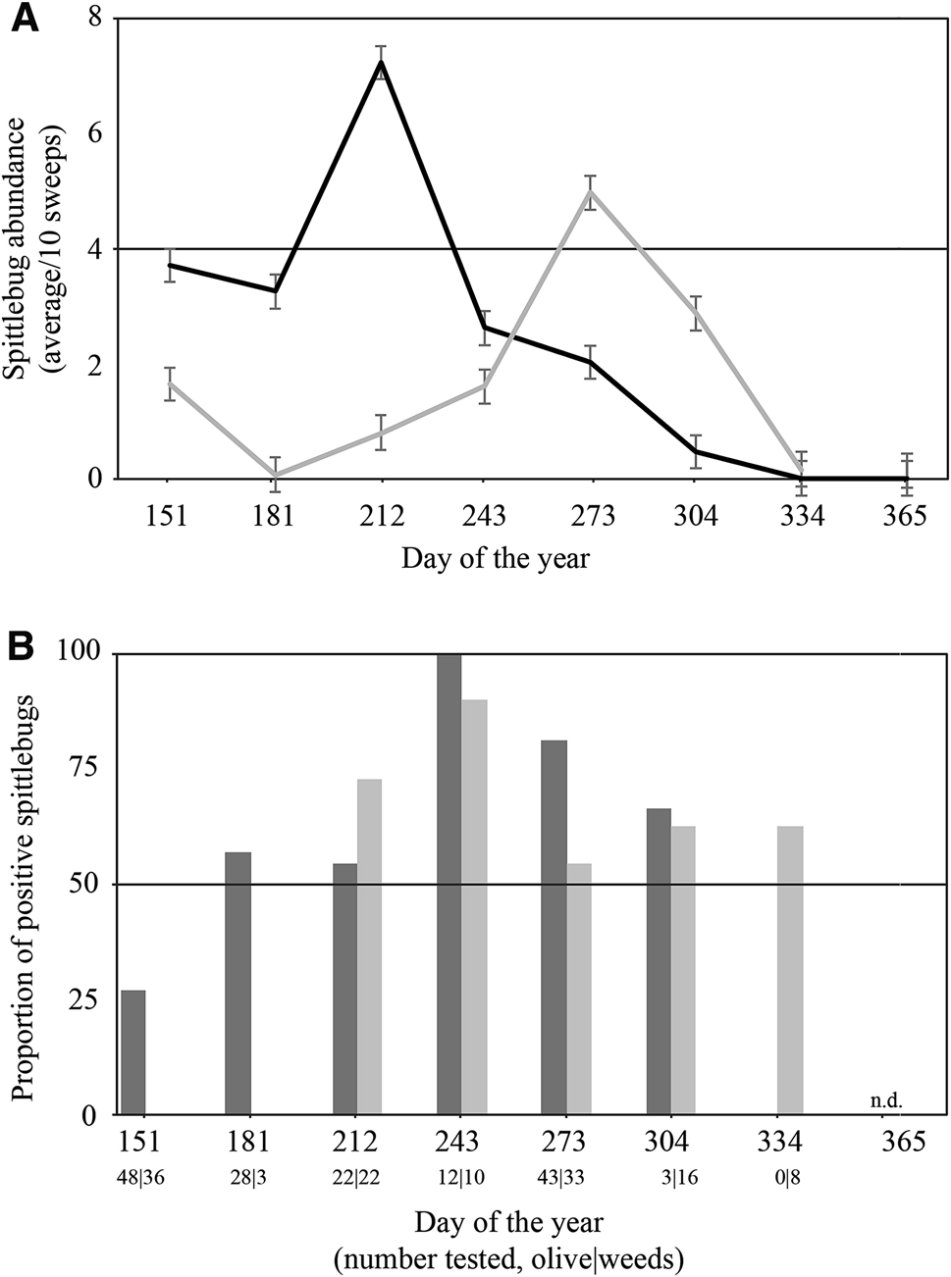
**a** Standardized densities (mean ± model-predicted SE) of *Philaenus spumarius* from May to December 2014 in olive trees (*black*) and weeds (*grey*) in Apulia, Italy; each sample corresponds to ten sweeps. **b** *P. spumarius X. fastidiosa* infection rate as determined by RT-PCR detection. *Numbers* below dates indicate the number of individuals that were tested from olive trees or weeds. SEs were calculated from ANOVA model results, predicted for each data point, because data on *P. spumarius* densities per replicate were lost and only means for each date were recovered. Model-predicted SE result in uniform *error bars*

Although no more than from three up to five individuals per collection date, adult *P. spumarius* were collected continuously during the 2013–2014 winter, even during the emergence of the first instar spittle masses in late February 2014. We observed numerous spittle masses on plants starting from the middle of March, both inside and outside cultivated orchards. *P. spumarius* adults were regularly collected by sweeping the olive canopy in May, June and July; in the same period, populations on weeds declined as the ground vegetation became drier (Fig. 2a). A reversal of this trend was observed starting from the end of July, with *P. spumarius* adults collected on plants of *Conyza sp.* and a reduction of the number of individuals on olive trees. Overall, densities of *P. spumarius*, calculated as the total number of individuals on the total number of bags collected on that host as described above, were not different between olives and weeds (*n* = 8, *F*_*1,7*_ = 0.356, *P* = 0.57).

From December 2013 to May 2014, none of the adults collected from weeds were positive for *X. fastidiosa*. During 2014, the first *P. spumarius* positive for *X. fastidiosa* were collected from olive canopies in the middle of May. The percentage of infected *P. spumarius* adults collected on olive and weeds was highest in August, but was over 50 % in all summer (Fig. 2b). Overall, the proportion of *P. spumarius* infected with *X. fastidiosa* (i.e., infection prevalence) was marginally greater in olives than in weeds (*n* = 8, *F*_*1,7*_ = 5.591, *P* = 0.050); infection prevalence in olives was about double that in weeds (Fig. 2b).

### *Philaenus spumarius* transmits *X. fastidiosa* from olive-to-olive

All the spittlebugs collected and tested before the experiment, and those not included in the experiment since death during the transfer to infected area, tested negative to *X. fastidiosa* by real-time PCR. Therefore, individuals used for the acquisition/inoculation experiment would have been likely free from the bacterium before the AAP. After the 4-day AAP on olive branches in the field, 16 *P. spumarius* and 6 *N. campestris* were found dead; of these 5 *P. spumarius* and 1 *N. campestris* individual tested positive for *X. fastidiosa*. At the end of the IAP, 11 of 35 *P. spumarius* and 1 of 16 *N. campestris* individuals were *X. fastidiosa*-positive. Two of four olive plants inoculated with *P. spumarius* were infected with *X. fastidiosa*, while none of the two olive plants inoculated by *N. campestris* tested positive (Table 3). Both olive and periwinkle plants kept as negative controls in the greenhouse remained *X. fastidiosa*-negative by real-time PCR.

### Impacts of host plant species on *X. fastidiosa* transmission efficiency by *P. spumarius*

**Fig. 3:**
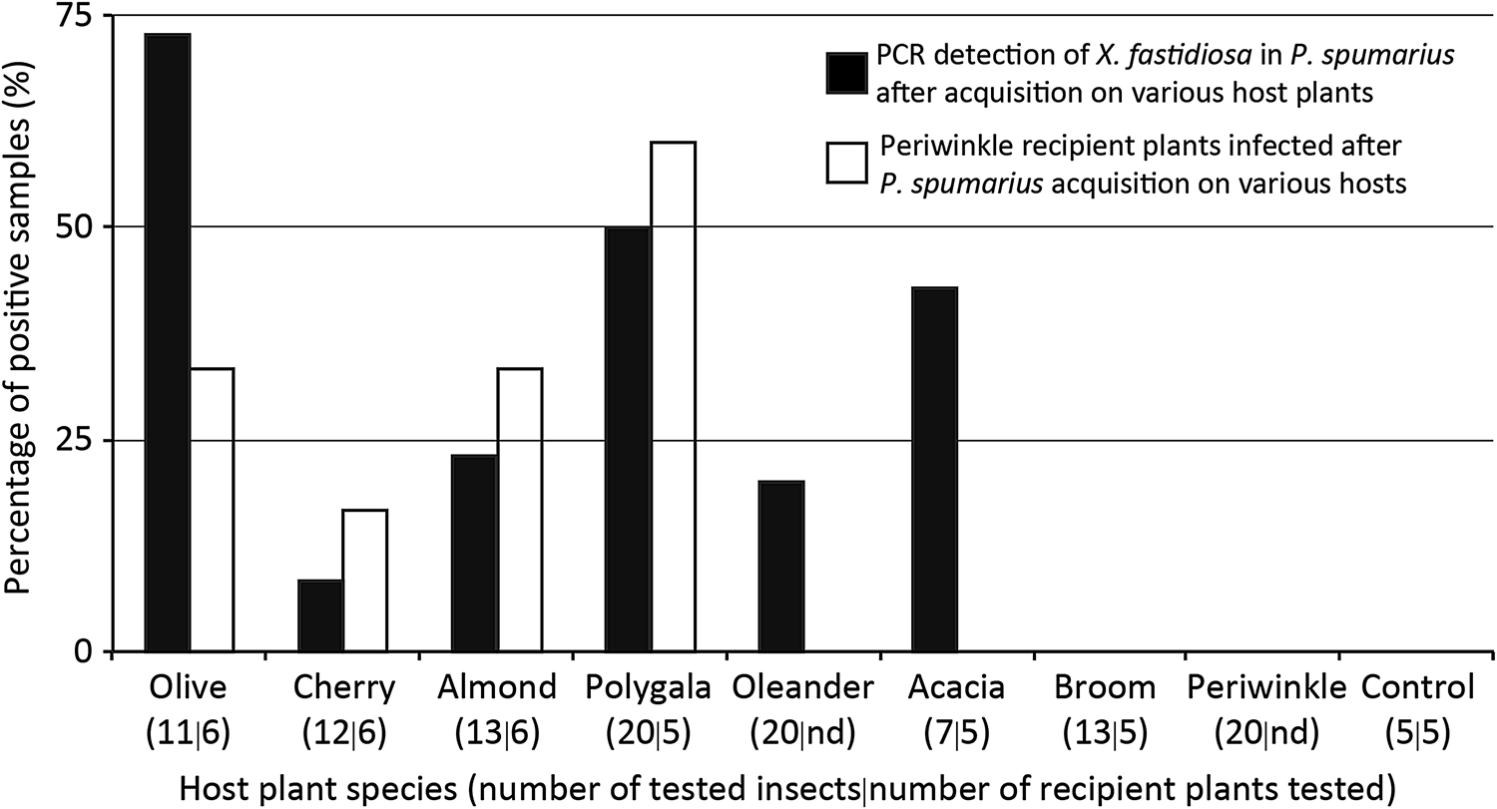
Percent of *Philaenus spumarius* acquiring *X. fastidiosa* from different host plant species (*black bars*) and percent of periwinkle plants inoculated with *X. fastidiosa* from infected *P. spumarius* (*white bars*). Numbers on the *x-axis* at the base of each pair of columns indicate the number of *P. spumarius* recovered from the source plant after AAP and tested by PCR (*left*) and the number of periwinkle plants inoculated with single spittlebugs randomly selected from those recovered after the IAP (*right*)

Acquisition efficiency under field conditions, as estimated by the percentage of *X. fastidiosa* real-time PCR-positive *P. spumarius*, was significantly different among pathogen source plant species (Fig. 3). Olive was the host with the highest percentage of positive individuals; acquisition efficiency from polygala and acacia were not statistically different from that from olive (Table 1). However, acquisition from broom, almond, cherry, oleander and periwinkle were all statistically lower than that from olive (Table 1). No acquisition occurred from the negative control (i.e., uninfected acacia). In contrast to acquisition, there were no significant differences in vector inoculation rates from various source plant species to periwinkle (Table 2).

**Table 1:** Statistical results from bias-corrected logistic regression testing differences between olive and other host plants in acquisition rate by *Philaenus spumarius* as estimated by PCR detection

Host plant	Estimate	SE^a^	χ^2^ statistic	*P* value
Intercept	0.89	0.66	2.148	0.143
Acacia (negative control)	−3.29	1.75	7.024	0.008**
Acacia (positive control)	−1.14	1.01	1.468	0.226
Broom	−1.99	0.92	5.657	0.017*
Almond	−4.18	1.64	14.740	<0.001***
Cherry	−2.92	1.12	9.865	0.002**
Oleander	−2.19	0.86	7.942	0.005**
Periwinkle	−4.60	1.61	19.460	<0.001***
Polygala	−0.89	0.80	1.388	0.239

The plant species name indicates the identity of the test plant

^a^ *SE* standard error of the coefficient estimate

* *P* < 0.05; ** *P* < 0.01; *** *P* < 0.001

**Table 2:** Statistical results from bias-corrected logistic regression testing differences between olive and other plant species as sources of *Xylella* *fastidiosa* for *Philaenus spumarius* followed by inoculation in periwinkle as a shared indicator host

Host plant	Estimate	SE^a^	χ^2^ statistic	*P*-value
Intercept	−0.59	0.85	0.579	0.447
Acacia (negative control)	−1.81	1.83	1.479	0.224
Acacia (positive control)	−1.81	1.83	1.479	0.224
Almond	0.00	1.20	0.000	1.000
Broom	−1.81	1.83	1.479	0.224
Cherry	−0.71	1.31	0.353	0.553
Polygala	0.92	1.24	0.670	0.413

The plant species name indicates the identity of the source plant

^a^ *SE* standard error of the coefficient estimate

### Scanning electron microscopy of the foregut of spittlebugs

**Fig. 4:**
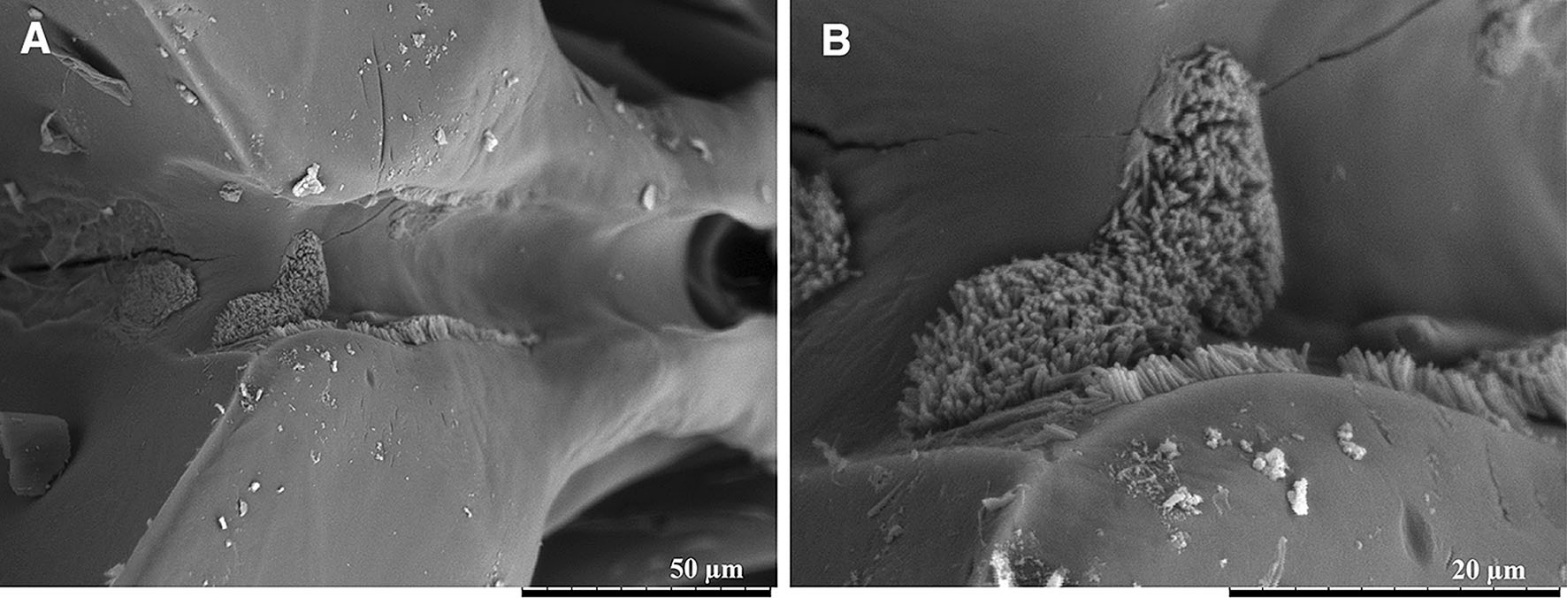
Scanning electron microscopy images of the **a** bacterial cells along the precibarium and cibarium of *Philaenus spumarius*; **b** details of bacterial cell aggregate at the distal area of the cibarium

Bacterial cells resembling *X. fastidiosa* were detected in two of the examined individuals (Fig. 4). We observed the cells lining the walls of the precibarium (Purcell et al. 1979; Brlansky et al. 1983; Almeida and Purcell 2006) and at the entrance of the cibarium along the groove of the floor of the pump chamber. Along the precibarium, the bacterial cells were attached polarly; cells were also noticed at the beginning of the food duct, distally to the precibarial valve. Moreover, sideways-attached cells (Almeida and Purcell 2006) were noticed on the precibarium, proximally to the cibarial pump floor (Table 3).

**Table 3:** Summary of transmission experiments with *Philaenus spumarius* and *Neophilaenus campestris* after a 4-day bacterial acquisition access period on *X. fastidiosa*-infected olive branches in the field

Recipient plant	*Neophilaenus campestris*		*Philaenus spumarius*		Infected plants
	Insects per plant (#)	*X. fastidiosa* infected (#)	Insects per plant (#)	*X. fastidiosa* infected (#)	
Olive	3	0			No
Periwinkle	4	0			No
Olive	3	0			No
Periwinkle			2	1	No
Olive			5	1	No
Olive			4	2	Yes
Olive			4	2	Yes
Olive			4	0	No

Insects were transferred to healthy olive and periwinkle recipient plants for a 4 days inoculation access period

## Discussion

The introduction of *X. fastidiosa* into Italy, and its primary role in olive desiccation and dieback as clearly shown by Saponari et al. (2016), has highlighted important knowledge gaps for the development of management practices aimed at reducing the spread of this pathogen in Europe. Among these is the limited information available on spittlebugs as *X. fastidiosa* vectors. In this study, we attempted to address questions of immediate importance, such as which potential vector species are present in olive orchards in southern Apulia. *P. spumarius* transmitted *X. fastidiosa* from olive to olive plants as well as from a range of infected-source plant species to periwinkle test plants. Furthermore, the spittlebug *P. spumarius* was the most abundant species found in orchards on both weeds and olive trees; *X. fastidiosa* prevalence in *P. spumarius* on olive trees was approximately twice that of insects collected from weeds, but the prevalence was very high in both environments. Although we cannot be completely sure that the insects used for acquisition from olive and different source plants were *X. fastidiosa*-free since they were field collected, despite the fact that all the insects tested by real-time PCR before the experiment were devoid of the bacterium, transmission by *P. spumarius* to olive plants together with the observed insect population shift and vector infectivity trend represents an important breakthrough in the understanding of the *X. fastidiosa* CoDiRO transmission biology. In summary, *P. spumarius* has to be considered an epidemiologically relevant vector species in Salento (southern Italy, Apulia).

Although there are several species of spittlebugs (Cercopoidea), cicadas (Cicadoidea) and sharpshooter leafhopper (subfamily Cicadellinae) throughout Europe (EFSA 2015), in Apulia only four potential vector species were collected in our surveys: three spittlebugs (*P. spumarius*, *N. campestris* and *C. sanguinolenta*) and one cicada (*C. orni*; this species was frequently observed during the summer but its prevalence and abundance were not determined in this study). Only *P. spumarius* was abundant and prevalent throughout the survey period, suggesting that it may be the most important vector in the region. However, because transmission efficiency (Daugherty et al. 2010), host plant preference throughout the year (Purcell 1981) and other factors are important in determining the epidemiological role of individual species, all potential vectors should be studied in the future so that their relative roles on disease spread will be better understood. This is especially true in the case of cicadas: there are only two reports of cicadas as *X. fastidiosa* vectors (Krell et al. 2007; Paiao et al. 2002), and more research on this topic must be done as cicadas are common on olive throughout the Mediterranean and are among the largest and most numerous insects within the habitat in which they occur (Pinto-Juma et al. 2005; Patterson et al. 1997). The abundance of *P. spumarius* on olive trees and weeds during the sampling period did not vary statistically. Given the single year of observation carried out with a single sampling method, we consider the data about *P. spumarius* host plant shifting and abundance presented in this work preliminary. In addition, only one survey method was used (i.e., sweep net). Purcell et al. (1994) suggested that a combination of sampling methods provides a more accurate estimation of abundance and movement of insects; these data are important for the understanding of the role of a vector in disease spread (Purcell et al. 1994; Irwin and Ruesink 1986).

The first finding of infective *P. spumarius* on olive trees in late spring 2014, whereas all the individuals previously collected on herbaceous plants within and outside the olive orchard had tested negative for *X. fastidiosa* by PCR, together with the gradual increase in the percentage of infective spittlebugs collected from olive canopy and suckers during the season suggests that this host serves as an important source of inoculum for pathogen spread. Furthermore, because *X. fastidiosa* is persistent in insect vectors including spittlebugs (Severin 1950), vectors may inoculate olive trees over an extended period of time. This phenomenon may enhance disease symptom expression, similar to what was observed in grapevines (Daugherty and Almeida 2009). In other words, multiple and independent infections events could lead to a reduction of the incubation period compared to that derived from a single infection (Daugherty and Almeida 2009). Therefore, it is possible that olive quick decline syndrome (OQDS) symptoms are enhanced by the incremental effects of a very large number of independent infections. This hypothesis must be tested as it may have important disease management consequences.

The role of host plant species on *X. fastidiosa* transmission by spittlebugs was tested with pathogen acquisition performed under field conditions. This approach has both positive and negative aspects, namely that biotic and abiotic factors affecting *X. fastidiosa* populations within plants are realistic, while there is no control on the variation of these parameters. *X. fastidiosa* vector acquisition efficiency is correlated to bacterial populations within grapevines (Hill and Purcell 1997), an observation that can be extended to various host plant species (Almeida et al. 2005); therefore, it is possible that the bacterial populations in these plant species are variable and the main driver of observed differences. However, it should be noted that vector behavior is also a component affecting *X. fastidiosa* transmission, as demonstrated when vector species on one plant, as well as host plant tissue within the same plant, significantly impacted *X. fastidiosa* transmission (Daugherty et al. 2010). It is not possible in this case to determine whether the differences in acquisition efficiency are based on bacterial populations within plants, which were not measured, or vector-plant interactions. Nevertheless, the data indicate that differences in acquisition efficiency exist based on host plant species and these should be studied within an epidemiological context. It was not surprising to see no difference among host plants when inoculation was considered as this was also previously observed with sharpshooter vectors (Lopes et al. 2009).

While *P. spumarius* is likely an important vector in Apulia, *N. campestris* and *E. lineolatus* have also been reported as capable of acquiring *X. fastidiosa* (Elbeaino et al. 2014). Our sample sizes for *N. campestris* and *E. lineolatus* were too small to assess infection rates precisely. *Neophilaenus campestris* is unlikely to be a critical vector in Apulia because it was present at low populations; nonetheless, our data do not rule out the potential in other nearby regions where populations may be larger. In the case of the phloem-sap feeder *E. lineolatus*, however, this species is not considered to be a potential vector (Redak et al. 2004; Almeida et al. 2005). The leafhopper was tested and reported in this article because Elbeaino et al. (2014) found *X. fastidiosa*-positive individuals during a survey in 2013. On the contrary, Saponari et al. (2014) reported no positive individuals out of 30 tested.

Altogether this study indicates that *P. spumarius* is a commonly found vector of *X. fastidiosa* in Salento. *P. spumarius* was capable of acquiring and inoculating *X. fastidiosa* from/to different host plants, and other hosts in the environment served as pathogen inoculum sources. Furthermore, we demonstrated that vectors transmit *X. fastidiosa* from infected olive plants in the field to test plants maintained in greenhouse conditions; these results are similar to those observed by Krugner et al. (2014) in California with sharpshooter vectors and two other subspecies of *X. fastidiosa*. *P. spumarius* was first shown to be a *X. fastidiosa* vector in the late 1940s (Severin 1950); the data presented here follow expectations based on what is known about *X. fastidiosa* transmission with sharpshooter vectors (Severin 1950; Almeida et al. 2005). It will be important to pursue detailed studies on the biology and ecology of *P. spumarius* in order to set up effective and environmentally acceptable vector control methods. However, given that the EFSA panel considers the removal of infected plants in a system-based approach as the only option to prevent further spread of the pathogen to new areas (EFSA 2016), controlling vectors alone will be useless if sources of *X. fastidiosa* are not also removed. The data presented here suggest that *X. fastidiosa*-infected olive plants are likely the main bacterium source within olive orchards, and *P. spumarius* seems a major driver for *X. fastidiosa* secondary spread. Our hypothesis is strengthened by recent fulfillment of Koch’s postulate and data from artificial inoculation of olive, besides other species, by Saponari et al. (2016). Nevertheless, more research efforts are urgently needed to shed light on the transmission biology of a bacterium that threatens the Italian and Mediterranean olive industry.

## Author contribution statement

Conceived and designed the experiments: DC, MS, DB, GPM, DB, FP. Performed the experiments: DC, MS, AdS, GL. Analyzed the data: ARZ, DC. Contributed reagents/materials/analysis tools: AdS, GPM, DB, FP, ARZ, RPPA. Wrote the paper: DC, ARZ, RPPA. All authors read and approved the manuscript.
